# Influence of the densities and nutritional components of bacterial colonies on the culture-enriched gut bacterial community structure

**DOI:** 10.1186/s13568-021-01240-6

**Published:** 2021-05-31

**Authors:** Yanrong Gu, Dong Yan, Minna Wu, Min Li, Puze Li, Jingjing Wang, Yahan Chang, Fan Yang, Shaojun Di, Shijun Ni, Mengjie Yang, Jieyu Liu

**Affiliations:** 1 Xinxiang Key Laboratory of Pathogenic Biology, Department of Microbiology, School of Basic Medical Sciences, Xinxiang Medical University, 601 Jinsui Road, Hongqi District, Xinxiang, 453003 Henan China; 2 Laboratory of Genetic Regulators in the Immune System, Henan Collaborative Innovation Center of Molecular Diagnosis and Laboratory Medicine, School of Laboratory Medicine, Xinxiang Medical University, Xinxiang, 453003 Henan China

**Keywords:** Culture-enriched, Gut bacterial community, Culture methods, Diversity, Nutritional components of media, Density

## Abstract

Isolating relevant microorganisms is still a substantial challenge that limits the use of bacteria in the maintenance of human health. To confirm which media and which bacterial colony densities can enrich certain kinds of bacteria, we selected eight common media and used them to enrich the gut microorganisms on agar plates. Then, we calculated the numbers of bacterial colonies and collected the bacterial culture mixtures from each kind of medium. Using the Illumina HiSeq platform, we analyzed the composition and diversity of the culture-enriched gut bacterial community. Our data suggested that medium supplemented with blood could increase the diversity of the bacterial community. In addition, beef powder and peptone could significantly change the culture-enriched bacterial community. A moderate density (100–150 colony-forming units per plate) was optimal for obtaining the highest diversity on the agar. Similarly, membrane transport was significantly enriched in the moderate-density group, which indicated a more active metabolism in this density range. Overall, these results reveal the optimal culture conditions, including the densities of colonies and nutritional components for various gut bacteria, that provide a novel strategy for isolating bacteria in a way that is targeted and avoids blinded and repetitive work.

**Supplementary Information:**

The online version contains supplementary material available at 10.1186/s13568-021-01240-6.

## Introduction

The important role of the gut microbiota in human health is becoming abundantly apparent. It is believed that the 1–2 kg of microorganisms in the human gut contain 150 times more genes than the human genome itself (Bäckhed et al. 2005; Patterson et al. 2016; Qin et al. 2010). The occurrence of many diseases, such as diabetes, obesity, and colorectal cancer, is related to the gut microbiota (Patterson et al. 2016; Qin et al. 2012; Wong and Yu 2019). In addition, the gut microbiota influence inflammation and immunity both locally and systemically (Abt et al. 2012; Clemente et al. 2012; Takeshi et al. 2011), and they have been demonstrated to contribute to cancer therapy (Iida et al. 2013). The development of omics technologies, beginning with metagenomics, highlighted the relationship between gut bacteria and human health. However, genomic technologies have provided a limited perspective as they cannot easily detect the minority populations (Lagier et al. 2012). A previous study has revealed that 80% of gut bacteria are unknown and considered unculturable (Lagier et al. 2018), which has made it difficult to further uncover the relationship between gut bacteria and disease. It is difficult to elucidate the role of microorganisms in human health without the ability to culture some microorganism. Therefore, because of their importance of unculturable microorganisms, the methods that isolate them have received more attention.

Culture methods for isolating unculturable microorganisms have been improved in previous studies. For in situ culture, the natural habitats of the targeted microorganisms could be mimicked. For example, Jung et al. developed a new technique, I-tip, which permitted microorganisms to grow utilizing chemical compounds in their natural environment, and it narrowed the gap between cultivated and uncultivated species (Jung et al. 2015). Furthermore, microbial interactions are important for population viability, and microorganisms can cooperate with each other by releasing metabolites and signaling molecules. Substances such as humic acid, signaling molecules, enzymes (for coping with reactive oxygen species), or inhibitors of undesired organisms (Alain and Querellou 2009; Leadbetter et al. 1999; Stevenson et al. 2004) were added to the medium, and more unculturable microorganisms were collected. In recent years, microbial culturomics has been a culturing approach that uses multiple techniques, such as MALDi-TOF mass spectrometry and 16S rRNA sequencing, for the identification of bacterial species. Using culturomics techniques, many microorganisms were isolated that had previously been considered unculturable (Lagier et al. 2016).

In this study, to ensure the consistency of fecal samples, we selected the commonly used mouse model BALB/c. We investigated how the nutritional components and densities influence the composition and diversity of the bacterial community, which bacteria could be enriched and the conditions of nutritional composition or density that allowed enrichment, and what the interaction was among bacteria at various densities. Thus, on the basis of these results, we determined a definite medium for the future isolation of a targeted bacterium in a certain density of bacterial colonies.

## Materials and methods

### Mice fecal samples collection

Three 6-week-old male BALB/c mice were purchased from Beijing Vital River Laboratory Animal Technology Co., Ltd. (Beijing, China). For environmental adaptation, mice were housed for 1 week before the experiment. Mice were housed in individually ventilated caging systems under a 12-h light/dark cycle at an environmental temperature of 23℃ ± 2℃ and a humidity of 55% ± 5%, and the mice had free access to sterilized standard rodent chow food and sterilized water. Three fecal samples were collected from each of the three mice. A 0.1 g sample of feces was resuspended in 1 mL of sterile saline and homogenized for 5 min using a vortex 10-diluted fecal suspension. Another 0.1 g of feces was stored in a − 80℃ refrigerator. After the study, all the mice were euthanized by cervical dislocation and subsequently treated as nonhazardous waste. Animal care was performed according to the National Institutes of Health’s Guide for the Care and Use of Laboratory Animals, and the experimental protocol was approved by the Institutional Animal Care and Use Committee of Xinxiang Medical University.

### Cultivation of fecal bacteria

For each 10^6^- and 10^7^-diluted fecal suspension, 100 μL was spread-plated onto eight kinds of medium plates, namely, modified Gifu anaerobic medium (GAM) (Haibo, China), brain–heart infusion medium (BH) (Haibo, China), chopped meat medium (CM) (Haibo, China), nutrient agar medium (NA) (Haibo, China), tryptic soy agar medium (TSA) (Haibo, China), reinforced Clostridial medium (RCM) (Haibo, China), Mueller–Hinton medium (MH) (Haibo, China), and blood medium (BL) (Huankai, China). As a control group, the fecal suspensions left were stored at − 80 ℃. The nutritional components of each medium are listed in Table 1. Eight replicates of each fecal sample were spread-plated onto each medium. Plates were then incubated anaerobically at 37℃ in an anaerobic workstation (10% CO_2_, 10% H_2_, and 80% N_2_). To keep the oxygen concentration below 0.1%, the oxygen concentration was detected every day. After 72 h of incubation, the number of colony-forming units (CFUs) was counted.

**Table 1:** The composition of each medium (g)

	GAM	RCM	BH	NA	CM	TSA	BL	MH
Peptone	28	10	21	10	30	20	10	17.5
Phosphate	2.5	0	2.5	0	5	2.5	0	0
Yeast extract powder	5	3	0	0	5	0	0	0
Beef powder	2	10	0	3	3	0	10	2
Beef liver extract powder	1.2	0	0	0	0	0	0	0
Beef brain extract powder	0	0	4	0	0	0	0	0
Beef heart extract powder	0	0	4	0	0	0	0	0
Soluble starch	0.3	1	0	0	2	0	0	1.5
L-Cysteine	0.15	0.5	0	0	0	0	0	0
Glucose	3	5	2	0	3	2.5	0	0
Serum powder	13.5	0	0	0	0	0	0	0
Sodium thioglycolate	0.15	0	0	0	0	0	0	0
Sodium chloride	3	5	5	5	0	5	5	0
Sodium acetate	0	3	0	0	0	0	0	0
Chopped meat	0	0	0	0	1	0	0	0
Defidrinated sheep’s blood	0	0	0	0	0	0	80 (mL)	0

### DNA extraction and PCR amplification

The plates with a single bacterial colony diameter that was greater than 1 cm were discarded. Thus, we collected the bacterial colonies in the plates of each medium and each dilution and added them into a 2 mL centrifuge tube using normal saline. The supernatant was discarded after centrifugation for 10 min at 7000*g*. Genomic DNA of bacterial colony mixtures and uncultured fecal samples (control group) was extracted using the Biomiga Stool gDNA Miniprep kit (Biomiga, USA) according to the manufacturer’s protocols. The V3-V4 region of the bacterial rRNA gene was amplified by PCR (98 °C for 1 min, followed by 30 cycles at 98 °C for 10 s, 50 °C for 30 s, 72 °C for 30 s and a final extension at 72 °C for 5 min) using the primers 338F (5′-ACTCCTACGGGAGGCAGCAG-3′) and 806R (5′-GGACTACHVGGGTWTCTAAT-3′) (Huws et al. 2007), where the barcode was a 6-base sequence unique to each sample. PCR amplification was conducted using Phusion High-Fidelity PCR Master Mix (New England Biolabs, USA). GeneJET Gel Extraction Kit (Thermo Scientific, USA). Amplicons were purified using the GeneJET Gel Extraction Kit (Thermo Scientific, USA).

### Illumina HiSeq sequencing

Sequencing libraries were generated using an Illumina TruSeq DNA PCR-Free Library Preparation Kit (Illumina, USA) following the manufacturer’s recommendations, and index codes were added. Finally, the qualified libraries were sequenced on an Illumina HiSeq2500 platform belonging to Novogene Co. Ltd (Beijing, China), which generated 250 bp paired-end reads.

### Processing of sequencing data and statistical analyses

The raw read sequences were demultiplexed, quality-filtered, and dereplicated using vsearch (version 2.8.1). The operational taxonomic units (OTUs) were clustered using vsearch based on the UNOISE algorithm (Edgar 2016). QIIME 2 was used to analyze α diversity, β diversity and the taxonomy of each 16S rRNA gene sequence against the SILVA (SSU132) 16S rRNA database (Bolyen et al. 2019). Alpha diversity was estimated by Shannon, Faith_PD, evenness, and the observed OTU indices using QIIME 2 (Bolyen et al. 2019). Canonical correspondence analysis (CCA) was conducted using R software (version 3.7.0) with the vegan package (Team 2018). The heatmaps were plotted using R software (version 3.7.0). The bar and line graphs were plotted using Origin software (version 8.0). Welch’s t tests were performed using the statistical analysis of metagenomics profiles software (STAMP) (Parks et al. 2014) to identify significantly different genera and predictive functions between groups. The network was generated using the CoNet plugin version 1.0b7 for Cytoscape v 3.6.0 based on the nonparametric Spearman correlation coefficients with a minimal cutoff threshold of ρ = 0.8 (*p* < 0.05, Bonferroni corrected) (Faust et al. 2012; Saito et al. 2012). The online Galaxy version of the phylogenetic investigation of communities by reconstruction of unobserved states (PICRUSt, http://huttenhower.org/galaxy/) was used to predict the metagenome function (Langille et al. 2013).

### Strain isolation using TSA and TSA supplemented with 8% defibrated sheep blood medium

To confirm the role of blood in the cultivation of fecal samples, bacterial isolates of fecal samples were identified and analyzed on TSA and TSA that were supplemented with 8% defibrated sheep blood medium. A 100 μL sample of each 10^5^-diluted fecal suspension was spread-plated onto the two kinds of media. Plates were then incubated anaerobically at 37℃ in an anaerobic workstation (10% CO_2_, 10% H_2_, and 80% N_2_). Three replicates of each fecal sample were spread-plated in each medium. After 72 h of incubation, 20 colonies in each plate were picked randomly and purified, but some colonies could not grow in the process of purification culture. Colony PCR amplification of the 16S rRNA gene was performed with methods described by Li et al. (Li et al. 2007). The purified PCR products were sequenced with an ABI PRISM automatic sequencer (model 3730XL). The partial 16S rRNA gene sequences (first 61–720 bp) were compared with the available 16S rRNA gene sequences from GenBank for identification using the BLAST program and a web-based tool at http://www.ezbiocloud.net as described by Kim et al. (Yoon et al. 2017).

## Results

### Medium changed the diversity and composition of the gut bacterial community

We obtained 4,291,005 combined raw reads and 1,848,155 valid reads after completing the demultiplexing and quality filtering. The valid reads were clustered into 606 unique OTUs.

**Fig. 1:**
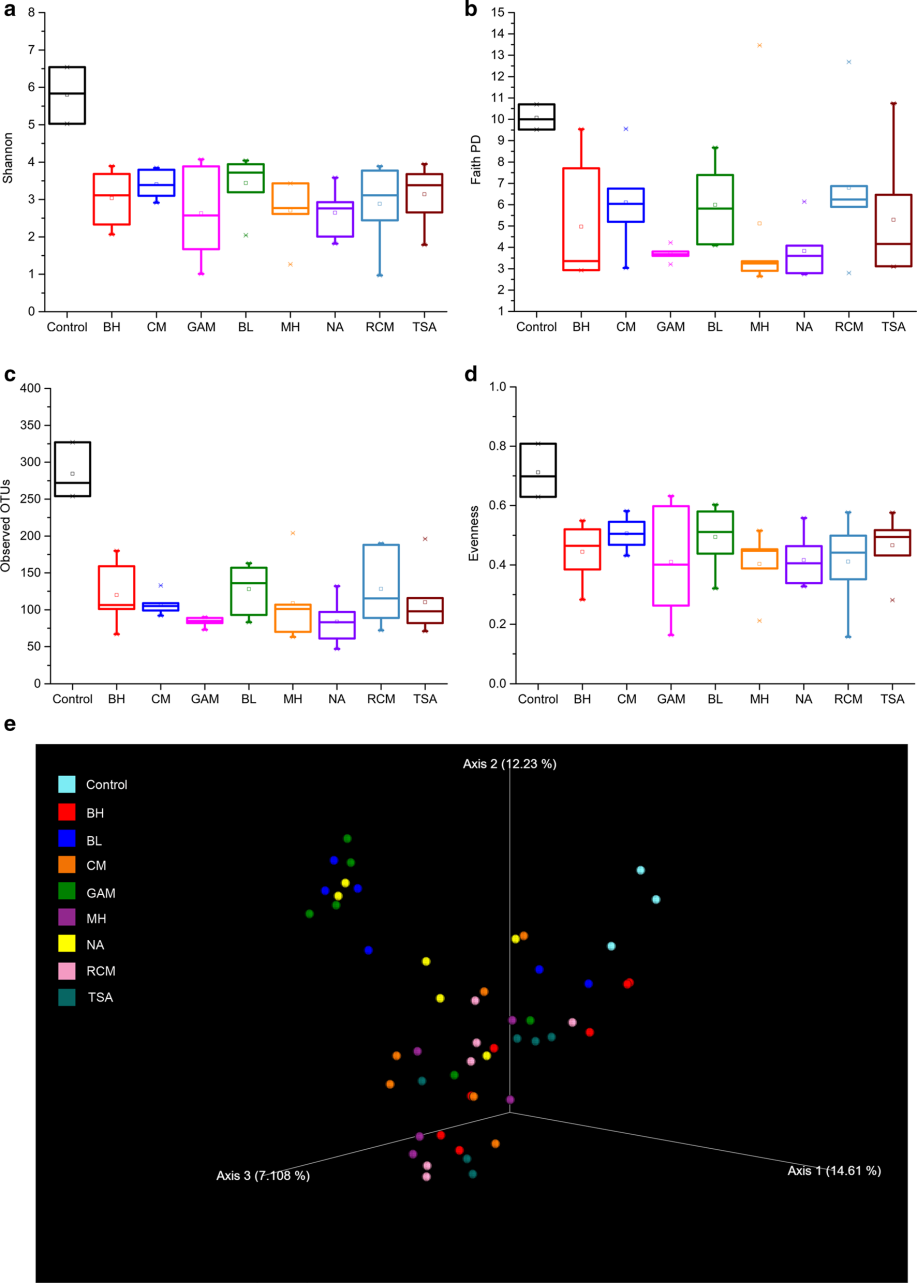
Diversity and composition of cultured gut bacterial communities in various media and uncultured feces. Shannon (**a**), faith pd (**b**), observed OTUs (**c**), and evenness index (**d**) in various media and uncultured feces. Boxes show medians and interquartile ranges (IQRs); whiskers denote lowest and highest values within 1.5 times the IQR from the first and third quartiles; outliers are shown as individual points. **e** Principal co-ordinates analysis (PCoA) of fecal cultured communities in various media and uncultured feces

The alpha diversity was estimated by the Shannon, Faith PD, observed OTUs, and evenness index. The rarefaction curves indicated that the high-throughput sequencing had captured the dominant phylotypes (Additional file 1: Fig. S1). As we expected, the diversities of the cultured gut bacterial community were significantly lower than those in uncultured feces. Based on the Shannon, observed OTUs, and evenness index results, the highest diversity and richness were observed in BL medium compared to other media. Furthermore, based on the Shannon and Faith PD index, we found higher diversity in the CM and RCM mediums and lower diversity and richness in the other media. These results indicated that the medium influenced the diversity of the gut bacterial community after the anaerobic cultivation (Fig. 1a–d).

We assessed the composition of the gut bacterial community using principal coordinate analysis (PCoA). As shown in Fig. 1e, the uncultured fecal composition of the gut bacterial community was clustered together, and the cultured fecal composition of the gut bacterial community was clustered together in BL, GAM, and NA medium and in BH, CM, MH, RCM, and TSA, which indicated that the medium influenced the composition of the gut bacterial community.

**Fig. 2:**
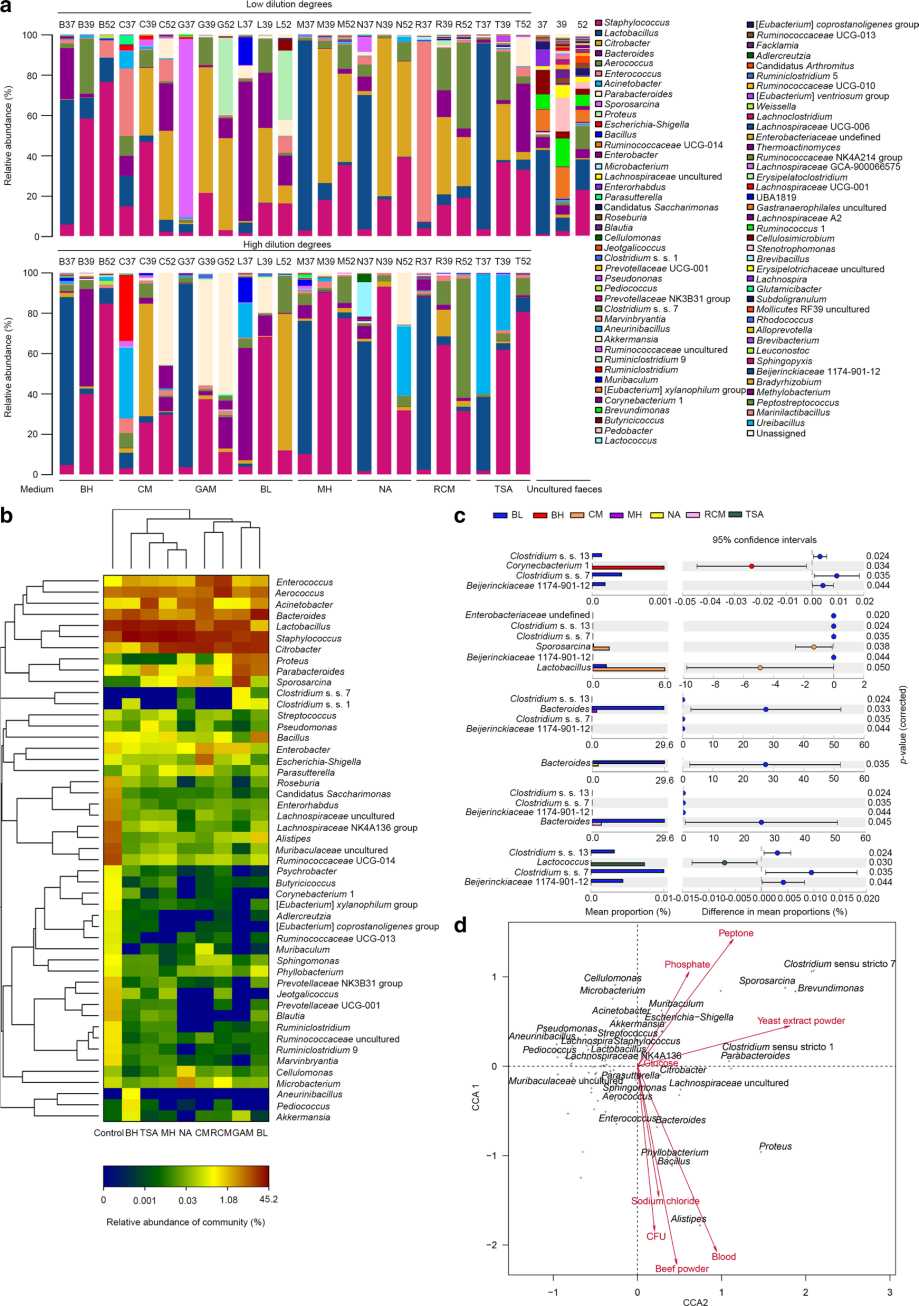
Differences in the relative abundances of genera affected by nutritional components. **a** Bar plot indicating the relative abundances of the dominant genera in each sample (< 0.0002%). **b** Heatmap analysis indicating the relative abundances of the dominant genera in various media (< 0.02%). **c** STAMP analysis indicated the genera that were significantly different between the BL medium and other media. **d** Canonical correspondence analysis (CCA) between the gut microbiota community and the related indices (peptone, blood, beef powder, sodium chloride, yeast extract powder, phosphate, glucose, and CFU). The bacteria shown in the panel are dominant genera (< 0.02%)

The bacterial community of the uncultured feces was dominated by *Lactobacillus* (21.2%), *Ruminococcaceae* UCG-014 (10.8%), *Muribaculaceae* uncultured bacterium (8.9%), *Staphylococcus* (8.7%), *Lachnospiraceae* NK4A136 group (6.7%), and *Alistipes* (6.2%). The bacterial community of the cultured feces was dominated by *Staphylococcus* (27.9%), *Lactobacillus* (18.3%), *Citrobacter* (16.3%), *Aerococcus* (7.5%), and *Bacteroides* (7.1%) (Fig. 2a, Additional file 2: Table S1). Large differences in the relative abundance of the genera were observed between the uncultured (Control) and cultured fecal samples using heatmap analysis; for example, *Ruminococcaceae* UCG-014, *Muribaculaceae* uncultured, *Alistipes*, *Lachnospiraceae* NK4A136 group, *Lachnospiraceae* uncultured, *Enterorhabdus*, Candidatus *Saccharimonas*, and *Parasutterella* were largely increased in the uncultured fecal samples and decreased in the cultured fecal samples, which indicated that these genera were hard to isolate in vitro; however, *Enterococcus*, *Aerococcus*, *Acinetobacter*, *Bacteroides*, *Lactobacillus*, and *Staphylococcus* showed similar abundances in the uncultured and cultured fecal samples, which indicated that these genera were easier to isolate in vitro (Fig. 2b). Importantly, we identified the significant genera from different media using statistical analysis of metagenomics profiles software (STAMP). Compared to the other media, more significant genera were observed in the BL medium. For example, *Bacteroides* was significantly increased in the BL medium compared to the MH, NA, and RCM mediums; *Corynecbacterium* 1, *Lactobacillus*, and *Lactococcus* were significantly increased in the BH, CM, and TSA, respectively, compared to the BL medium (Fig. 2c). Therefore, the results suggested that nutritional preferences changed the gut bacterial community on the agar plates.

### The concentration of beef powder, defibered sheep blood, yeast extract powder, and peptone and the number of colony-forming units (CFUs) significantly influenced the composition of the gut bacterial community

The nutritional components of each medium are listed in Table 1. Cluster and correlation analysis based on the nutritional components of media showed the differences in the media. The BL medium was clustered into a single group, the NA and RCM medium were clustered into another group, and the GAM, CM, MH, BH, and TSA medium were clustered into a third group (Additional file 1: Fig. S2). Furthermore, we used canonical correspondence analysis and permutation tests (Table 2) to investigate which main nutritional components in the medium significantly influenced the bacterial composition (CCA, Fig. 2d). CCA showed that beef powder and defibered sheep blood were highly significantly correlated with the composition of the bacterial community (P < 0.01); yeast extract powder and CFU were significantly correlated with the composition of the bacterial community (P < 0.05). The beef powder, blood, and CFU showed similar patterns of impacting the composition of the bacterial community, which correlated positively with *Alistipes*, *Proteus*, *Bacillus*, *Phyllobacterium*, *Bacteroides*, and *Enterococcus*. The yeast extract powder and peptone were positively correlated with *Clostridium* sensu stricto 7, *Brevundimonas*, *Sporosarcina*, *Clostridium* sensu stricto 7, *Parabacteroides*, *Muribaculum*, etc. These results indicated that the concentration of these components and the number of CFUs influenced the composition of the cultured bacterial community.

**Table 2:** Permutation test of Canonical correspondence analysis

	CCA1	CCA2	r^2^	P
CFU	0.2032	− 0.9791	0.1596	0.038*
Peptone	0.6882	0.7255	0.1523	0.022*
Phosphate	0.5486	0.8361	0.0671	0.212
Yeast extract powder	0.9895	0.1449	0.1842	0.011*
Beef powder	0.3088	− 0.9511	0.2429	0.004**
Glucose	0.9910	0.1341	0.0002	0.993
Blood	0.5165	− 0.8563	0.2581	0.005**
Sodium chloride	0.2728	− 0.9621	0.1030	0.076

Significance: **P < 0.01, *P < 0.05

To confirm the role of blood in the cultivation of fecal samples, bacterial isolates of fecal samples on TSA medium with and without 8% defibrated sheep blood were identified and analyzed. Forty strains, which belong to *Lactobacillus* (95%), *Staphylococcus* (2.5%), and *Bifidobacterium* (2.5%) (Table 3), were isolated, purified, and sequenced from the TSA medium. Twenty-nine strains, which belong to *Lactobacillus* (65.5%), *Parabacteroides* (20.7%), *Bacteroides* (6.9%), *Phocaeicola* (3.4%), and *Parasutterella* (3.4%) (Table 4), were isolated, purified, and sequenced from the TSA medium with 8% defidrinated sheep blood. These results indicated that supplementation with defidrinated sheep blood increased the diversity and structure of the culture-enriched gut bacterial community. For example, *Bacteroides* were isolated from the TSA medium with defidrinated sheep blood.

**Table 3:** Strain information of isolates from TSA medium

Top-hit taxon	Top-hit strain	Similarity (%)	Numbers of strains
*Lactobacillus murinus*	NBRC 14221	100	29
*Lactobacillus taiwanensis*	DSM 21401	99.85	9
*Staphylococcus hominis* subsp. hominis	DSM 20328	99.7	1
*Bifidobacterium animalis* subsp. animalis	ATCC 25527	100	1

**Table 4:** Strain information of isolates from TSA medium with 8% defidrinated sheep’s blood

Top-hit taxon	Top-hit strain	Similarity (%)	Numbers of strains
*Lactobacillus murinus*	NBRC 14221	100	19
*Phocaeicola vulgatus*	ATCC 8482	99.55	1
*Parabacteroides distasonis*	ATCC 8503	98.33	1
*Parabacteroides distasonis*	ATCC 8503	98.48	2
*Parabacteroides distasonis*	ATCC 8503	98.18	3
*Bacteroides faecichinchillae*	JCM 17102	100	1
*Bacteroides xylanisolvens*	XB1A	99.7	1
*Parasutterella excrementihominis*	YIT 11859	99.7	1

### Density of bacterial colonies influenced the diversity and composition of the gut bacterial community

**Fig. 3:**
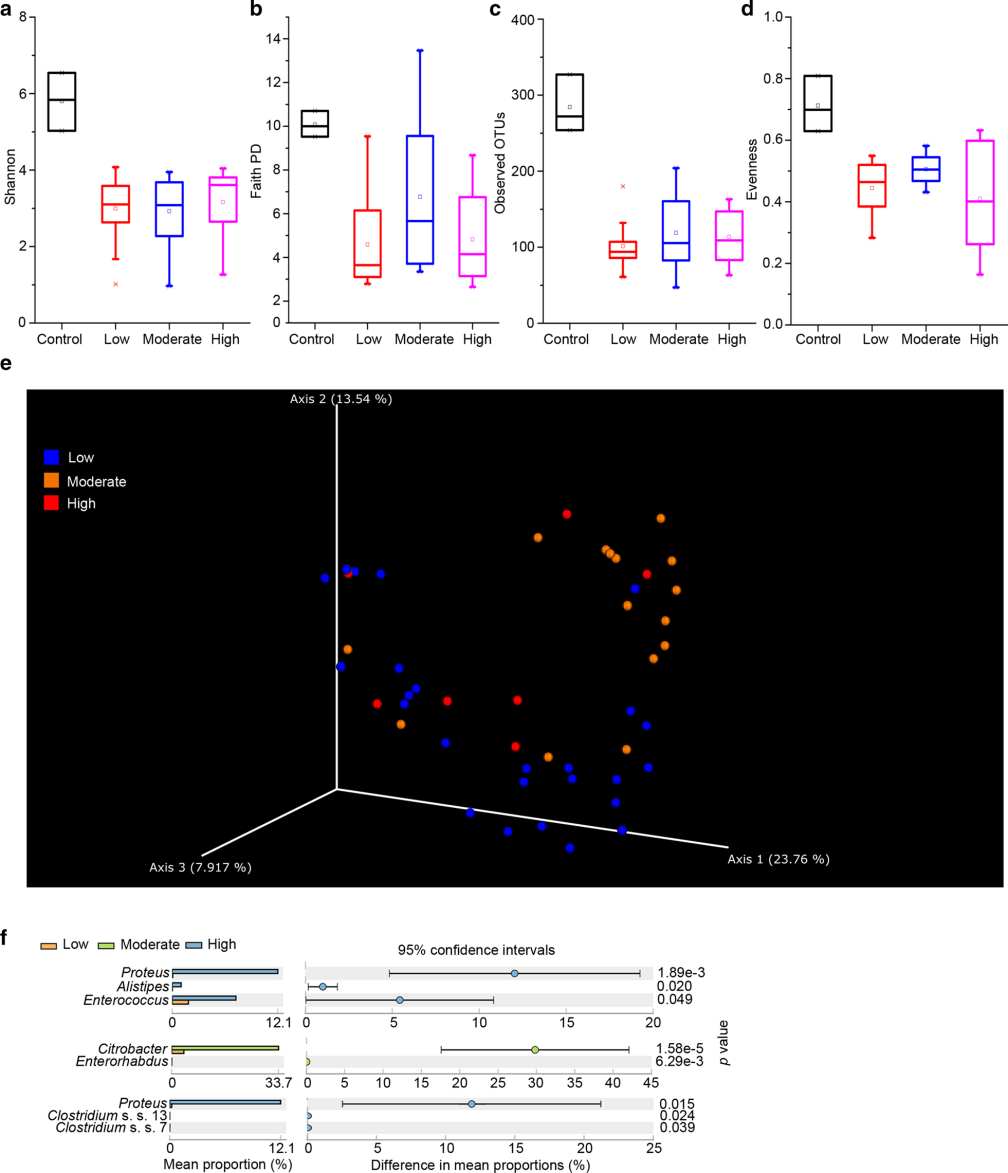
Density of bacterial colony affected the diversity and composition of the fecal bacterial communities. Shannon (**a**), faith pd (**b**), observed OTUs (**c**), and evenness index (**d**) in the low-, moderate-, and high-density groups and uncultured feces. **e** PCoA based on bray–curtis distance of the fecal cultured communities in the low-, moderate-, and high-density group and uncultured feces. **f** STAMP analysis indicated the genera that were significantly different between the low-, moderate-, and high-density groups

The number of CFUs influenced the composition of the cultured bacterial community, which suggested that the density of bacterial colonies was a key factor influencing the bacterial community on the agar. According to the numbers of CFUs, we classified the cultured fecal samples into low- (1–50 CFUs/plate), moderate- (51–150 CFUs/plate), and high-density (> 151 CFUs/plate) groups (Additional file 2: Table S2). A higher Faith PD and evenness index were observed in the moderate-density samples, yet no significant differences were observed in the Shannon and observed OTU index, which indicated that moderate density increased the diversity and evenness (Fig. 3a–d).

PCoA indicated that low-density and moderate-density samples clustered separately (Fig. 3e). Thus, we assessed the significant genera in the low-, moderate-, and high-density groups using STAMP. *Proteus*, *Alistipes*, and *Enterococcus* were significantly increased in the high-density group compared to the low-density group; *Citrobacter* and *Enterorhabdus* were increased in the moderate-density group compared to the low-density group; and *Proteus*, *Clostridium* s.s. 13, and *Clostridium* s. s. 7 were increased in the high-density group compared to the moderate-density group (Fig. 3f).

### Bacterial interactions among the gut bacterial community on agar plates

**Fig. 4:**
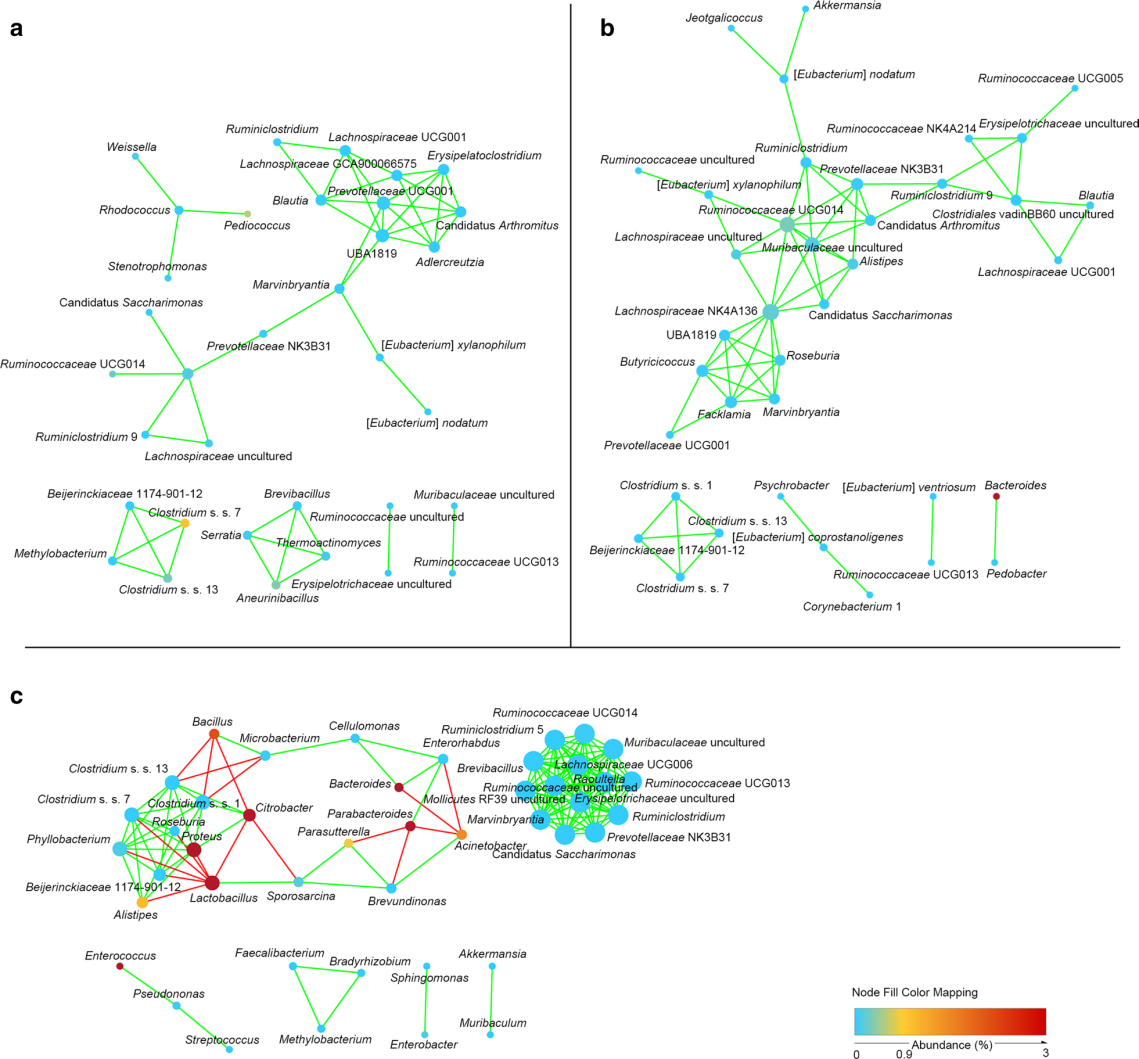
Network analysis indicating interactions among the genera in the low- (**a**), moderate- (**b**), and high-density groups (**c**). Each node is a bacterial genus, and the edges indicate significant co-occurrent (green) or mutual exclusion (red) interactions

To compare the possible interactions among the low-, moderate-, and high-density samples on the agar plates, the 98 genera we detected were used to construct three networks. In the resulting network, each node was a bacterial genus, and the edges indicated significant co-occurrent (green) or mutual exclusion (red) interactions (Fig. 4). The greatest number of nodes (44 nodes) and edges (151 edges) were observed in the high-density samples (Table 5). Furthermore, mutual exclusion interactions (18 edges) were only found in the network of high-density samples, which indicated that a high density of bacterial colonies could promote the interactions among the bacterial community and it might be more possible for them to inhibit bacterial growth.

**Table 5:** Network properties in low-, moderate-, and high-density samples

	Numbers of edges	Numbers of nodes	Average path length	Clustering coefficient	Heterogeneity
Low-density samples	50	34	2.84	0.47	0.60
Moderate-density samples	67	38	3.20	0.51	0.66
High-density samples	151	44	1.93	0.67	0.68

Topological properties are always used in network analyses to describe the complex pattern of interrelationships. High-density samples have the shortest average path length (average network distance, 1.93) and the highest clustering coefficient (the degree to which they tend to cluster together, 0.67), which indicated that high-density samples had the stronger interaction between the nodes (Table 5). *Prevotellaceae* UCG001 (7 edges) and UBA1819 (7 edges) were the key genera in the low-density samples (Fig. 4a). *Lachnospiraceae* NK4A136 (10 edges), *Ruminococcaceae* UCG014 (9 edges), and *Muribaculaceae* uncultured bacterium (8 edges) were the key genera in the moderate-density samples (Fig. 4b). The *Muribaculaceae* uncultured bacterium (13 edges), *Raoultella* (13 edges), *Ruminococcaceae* UCG014 (13 edges), *Mollicutes* RF39 uncultured (13 edges), *Erysipelotrichaceae* uncultured (13 edges), *Ruminiclostridium* (13 edges), *Ruminiclostridium* 5 (13 edges), *Marvinbryantia* (13 edges), *Brevibacillus* (13 edges), *Prevotellaceae* NK3B31 (13 edges), Candidatus *Saccharimonas* (13 edges), *Ruminococcaceae* UCG013 (13 edges), *Lachnospiraceae* UCG006 (13 edges), and *Ruminococcaceae* uncultured (13 edges) bacterium were the key genera with the most co-occurrent interactions, and *Lactobacillus* (7 edges) was the key genus with mutual exclusion interactions in the high-density samples (Fig. 4c).

### Functional prediction under different media and densities of bacterial colonies

**Fig. 5:**
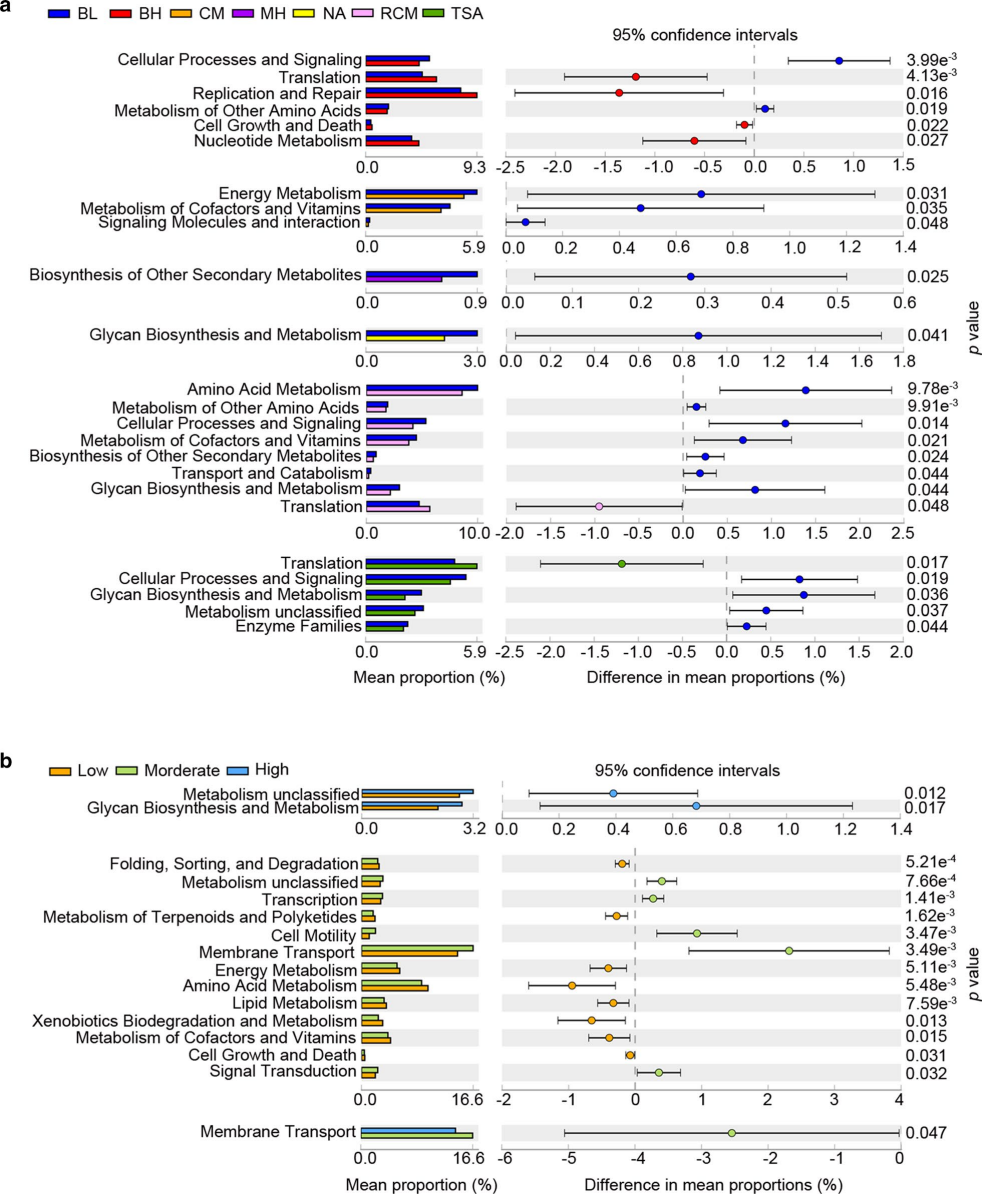
STAMP analysis indicated the differences of predicted functions in various media (**a**) and the densities of bacterial colonies (**b**)

STAMP was applied to further investigate whether the potential functions of gut microbiota on agar plates are typically associated with the nutritional components of medium and the densities of bacterial colonies, and it compared the relative abundance values of the KEGG pathways predicted by the phylogenetic investigation of communities by reconstruction of unobserved states (PICRUSt). To infer the effects of the nutritional components on the functions of microbiota in various media, we examined the L2 metabolic pathways with significant differences among the medium groups (Fig. 5a). Most of the metabolic pathways increased in the BL medium, for example, the cellular processes and signaling pathway was increased compared to that in the BH, RCM, or TSA medium; the biosynthesis and metabolism of glycan was increased compared to that in the NA, TSA, or MH medium; metabolism of the cofactors and vitamins was increased compared to in the CM, TSA, or RCM medium; energy metabolism was increased compared to that in the CM medium; biosynthesis of other secondary metabolites was increased compared to in the MH medium; and amino acid metabolism was increased comparing to in the RCM medium. Therefore, compared to other the media, the bacterial growth and metabolic processes, such as cellular processes and signaling, glycan biosynthesis and metabolism, and metabolism of cofactors and vitamins, were more active in the BL medium,.

Next, we assessed the effects of density on the functions that had significant differences (Fig. 5b). More functions with significant differences were observed between moderate- and low-density samples. For example, a moderate density promoted membrane transport and cell motility and inhibited amino acid metabolism and xenobiotic biodegradation and metabolism. Compared to the low-density samples, the high-density samples promoted glycan biosynthesis and metabolism. Furthermore, a moderate density promoted membrane transport compared to both the high- and low-density samples. Above all, the nutritional components of the medium and the bacterial colonies densities both changed some predictive functions, which may further influence the viability and growth of bacteria.

## Discussion

The process of isolating more microorganisms with the development of new culture techniques always takes a great amount of effort and time. Moreover, current knowledge is limited on which factors influence the composition and diversity of the cultured gut bacterial communities, and previous studies have focused on blindly obtaining many repeated bacterial colonies using culture methods. To explore a new method of isolating hard-to-culture bacteria, we selected eight common media to enrich gut bacteria on agar plates. Thus, we collected bacterial cultures in various media and analyzed the cultured gut bacterial community using high-throughput sequencing. The results indicated that the different nutritional preferences and densities of the bacterial colonies shaped the various bacterial communities, which suggested that the nutritional components and densities can be adjusted to isolate targeted gut microbiota.

A decrease in gut bacterial diversity was observed after in vitro anaerobic cultivation, which showed there were differences between the in vitro and in vivo environments. Most of the genera enriched in uncultured feces are unculturable bacteria. However, the known genera *Alistipes*, *Enterorhabdus*, and *Parasutterella* were also increased in uncultured feces. According to the description of these three genera, they are obligate anaerobic bacteria, and they can be described as facultative anaerobic bacteria that grow rapidly on agar (Clavel et al. 2009; Nagai et al. 2009; Rautio et al. 2003). This is a possible reason why they are hard to cultivate on agar. In particular, to date, only two and three species have been identified in *Enterorhabdus* and *Parasutterella*, respectively.

The medium is the major factor influencing bacterial growth. Culture collections (such as www.atcc.org or www.dsmz.de) recommend species-specific and complex-undefined media for growing individual gut bacteria. Eight common complex-undefined media were used in this study. The bacterial community in BL medium showed a higher diversity compared with the other media. Lagier et al. summarized the 18 optimal culture conditions and identified blood culture bottles, rumen fluid, and sheep blood as the three key nutrient substrates for growing bacteria; the addition of sheep blood increased the isolation rates of the new species, which also indicated the importance of blood in culturing gut bacteria (Lagier et al. 2015, 2016). Furthermore, *Bacteroides* was enriched significantly in the BL medium, which indicated that it could be isolated in the BL medium. This result is consistent with other studies, and some novel *Bacteroides* species were isolated using medium containing blood (Bakir et al. 2006a, b; Kitahara et al. 2012; Saputra et al. 2015). Interestingly, 692 unique anaerobic episodes were isolated from the blood cultures in a clinical study, while the *Bacteroides* spp. accounted for the most colonies (266/692, 38%) (Rassolie and Özenci 2019). *Lactobacillus* was significantly decreased in the BL medium compared to all the other media and uncultured feces. Therefore, it is suggested that the BL medium is not suitable for the growth of *Lactobacillus*. Moreover, *Lactobacillus* is the most common bacteria isolated from feces as their strong lactic acid production ability allow them to restrain the growth of other bacteria (Merino et al. 2019; Wasfi et al. 2018; Benmouna et al. 2020), which explains the higher diversity in the BL medium. The results above showed the advantages of the BL medium in cultivating various gut bacteria, while other media can be selected to isolate specific bacteria. For example, we can isolate *Aneurinibacillus*, *Pediococcus*, and *Akkermansia* using BH medium, *Microbacterium* using NA medium, *Muribactulum* using CM medium and so on.

The differences in cultured gut bacterial communities in various media are due to the influence of the nutritional components on the growth of bacteria. According to the clustering results that were based on the nutritional components of the medium, the BL medium was clustered into a single group. The CCA results showed that blood significantly influenced the cultured gut bacterial community. Many blood constituents play important roles in bacterial growth. For example, heme, an essential component of red blood cells, is indispensable for the *Porphyromonas gingivalis* growth activities (Champagne et al. 2007; Cueno et al. 2014). In contrast, excess heme concentration can be harmful (Lewis 2010; Olczak et al. 2005). For example, many cultured gut bacteria, such as *Lactobacillus leichmannii,* cannot grow without vitamin B12 supplementation (Kirmiz et al. 2020). Beef powder and peptone both significantly changed the cultured bacterial community, which indicated they have important roles in bacterial growth on agar. Kajihara et al. suggested that the growth rate of *Megasphaera elsdenii* on KMI medium with a higher ratio of beef extract to peptone was faster than that of other intestinal bacteria (Kajihara et al. 2017). Furthermore, we also observed that these two nutritional components had the opposite effects on bacterial growth. For example, the addition of peptone showed a positive correlation with the relative abundance of *Cellulomonas*, *Microbacterium*, etc., while beef powder showed a negative correlation with them. Therefore, determining which nutritional component can enrich which bacteria can provide the basis for adjusting the ratio and concentration of the nutritional components for cultivating the targeted bacteria.

Apart from nutritional components, bacterial interactions are another factor that influences the cultured bacterial community. The number of CFUs significantly influenced the bacterial community (Fig. 2, Table 2). Further analysis revealed that the densities of bacterial colonies have an important effect on the diversity and composition of the bacterial community. A higher diversity of the cultured bacterial community was shown in the moderate-density group, which indicated that too many or too few of the colonies had reduced the bacterial growth on the agar. To survive in complex surrounding communities, bacteria must cooperate and compete with each other (Nadell et al. 2016). Based on our network analysis, when the densities of colonies were low or moderate on the agar, the bacteria tended to cooperate with each other. Compared to the low-density group, more cooperating interactions were observed in the moderate-density group, which indicated that the space among the bacterial colonies decreased the cooperating interactions. Cooperation is widely found in bacterial communities, and bacterial communities have cooperative traits, such as diffusible public goods (Harrison et al. 2008). Products that increase individual fitness can also be shared between all members of a population (MacLean 2008). We observed more negative interactions in the high-density group, which suggested that the higher densities caused more competition among the bacterial community. Previous studies reported that bacteria could interact antagonistically by releasing toxins to affect competitors (Bottery et al. 2019; Granato et al. 2019). These toxins were diffusible or delivered toxic effector proteins between the toxin-producing cell and its target cell (Hayes et al. 2010; Sassone-Corsi et al. 2016). Therefore, we can isolate the strain that we wanted by adjusting the inoculation density. For instance, the *Citrobacter* species can be isolated with a moderate density, the *Proteus* species with a higher density, etc. In addition, network analysis can also guide the isolation of bacteria. We can try to add the fermentation solution of cooperating bacteria to promote the growth of specific bacteria that are difficult to cultivate. In this way, we can obtain more bacteria that we have not yet cultivated before.

Furthermore, we used functional analysis to understand the biochemical processes of bacteria in different culture conditions. For example, DNA replication and repair, translation, nucleotide metabolism, cell growth and death were enhanced in the BH medium, which implied that nutritional components attributed to the growth of bacteria and metabolism tended to be vigorous compared to the BL medium. Metabolism, including energy, cofactors and vitamins, glycans, secondary metabolites, etc., was enhanced in the BL medium, indicating that the BL medium provided rich nutrition for bacterial growth and metabolism. In addition, the density of the bacterial colonies also causes differences in the bacterial functions. Surprisingly, the greatest differences were observed between the moderate- and low-density groups and not between the high- and low-density groups. It is suspected that competing interactions kill more bacteria and make the functional activity of bacteria in the high-density group similar to that in the low-density group. Membrane transport was significantly enriched in the moderate-density group compared to both the low- and high-density groups. Transport proteins that have sensory purposes in the downstream activation of two-component systems have been described in several bacterial species. For example, the presence of bacitracin is sensed via the activity of the ABC transporter BceAB of *B. subtilis*. The external and cytoplasmic levels of the antibiotic bacitracin influence the activity of the ABC transporter protein BceAB and increase the transcription of transporter genes and other related functions (Alvarado et al. 2020; Fritz et al. 2015; Radeck et al. 2017). These studies confirmed the potential pathway by which density influences the function of membrane transport.

In conclusion, our data suggest that both the nutritional components and densities of bacterial colonies influences the gut bacterial community on agar plates. Medium supplementation with blood can increase the diversity of the bacterial community. In addition, beef powder and peptone are important factors that can significantly change the culture-enriched bacterial community. We observed that a moderate density (100–150 CFUs per plate) was optimal for ensuring a higher diversity on the agar. Similarly, the function of membrane transport was significantly enriched in the moderate-density group, which indicated a more active metabolism on the agar plates with this density range. Our work elucidated the optimal culture conditions, including the densities of colonies and nutritional components for various gut bacteria, which provides a novel strategy for the targeted isolation of bacteria that avoids blindness and repetitive work. Furthermore, many competing and cooperative interactions were observed among the culture-enriched bacterial communities, which will encourage more researchers to isolate uncultured bacteria by supplementation with culture that is filtrated with one single or mixed specific bacteria in the medium. In the future, the mechanism by which nutrient components regulate bacterial growth and interactions will require further study.

## Supplementary Information

**Additional file 1: Figure S1**. Rarefaction curves in all samples. **Figure S2**. Correlation and cluster analysis of nutritional components in medium based on Spearman correlation coefficient.

**Additional file 2: Table S1**: Relative abundance of fecal bacteria in genus level. **Table S2**: The numbers of CFU in various media (CFU/plate).

## Data Availability

The data generated or analyzed during this study are included in this published article and its Additional file. The raw reads were deposited into the NCBI Sequence Read Archive (SRA) database under accession number SRP249932.

## Abbreviations

PCoA: Principle coordination analysis
STAMP: Statistical analysis of metagenomics profiles software
CFU: Colony-forming unit
CCA: Canonical correspondence analysis
PICRUSt: Phylogenetic investigation of communities by reconstruction of unobserved states
GAM: Modified Gifu anaerobic medium
BH: Brain–heart infusion medium
CM: Chopped meat medium
NA: Nutrient agar medium
TSA: Tryptic soy agar medium
RCM: Reinforced clostridial medium
MH: Mueller–Hinton medium
BL: Blood medium
OTUs: Operational taxonomic units
