# Supplementary material for: Influence of the densities and nutritional components of bacterial colonies on the culture-enriched gut bacterial community structure
Source: AMB Express. 2021 May 31;11:78. doi: 10.1186/s13568-021-01240-6 (PMC8167003; doi:10.1186/s13568-021-01240-6)
Supplement: Supplementary file 1 — Additional file 1: Figure S1. Rarefaction curves in all samples. Figure S2. Correlation and cluster analysis of nutritional components in medium based on Spearman correlation coefficient. [file 13568_2021_1240_MOESM1_ESM.docx]

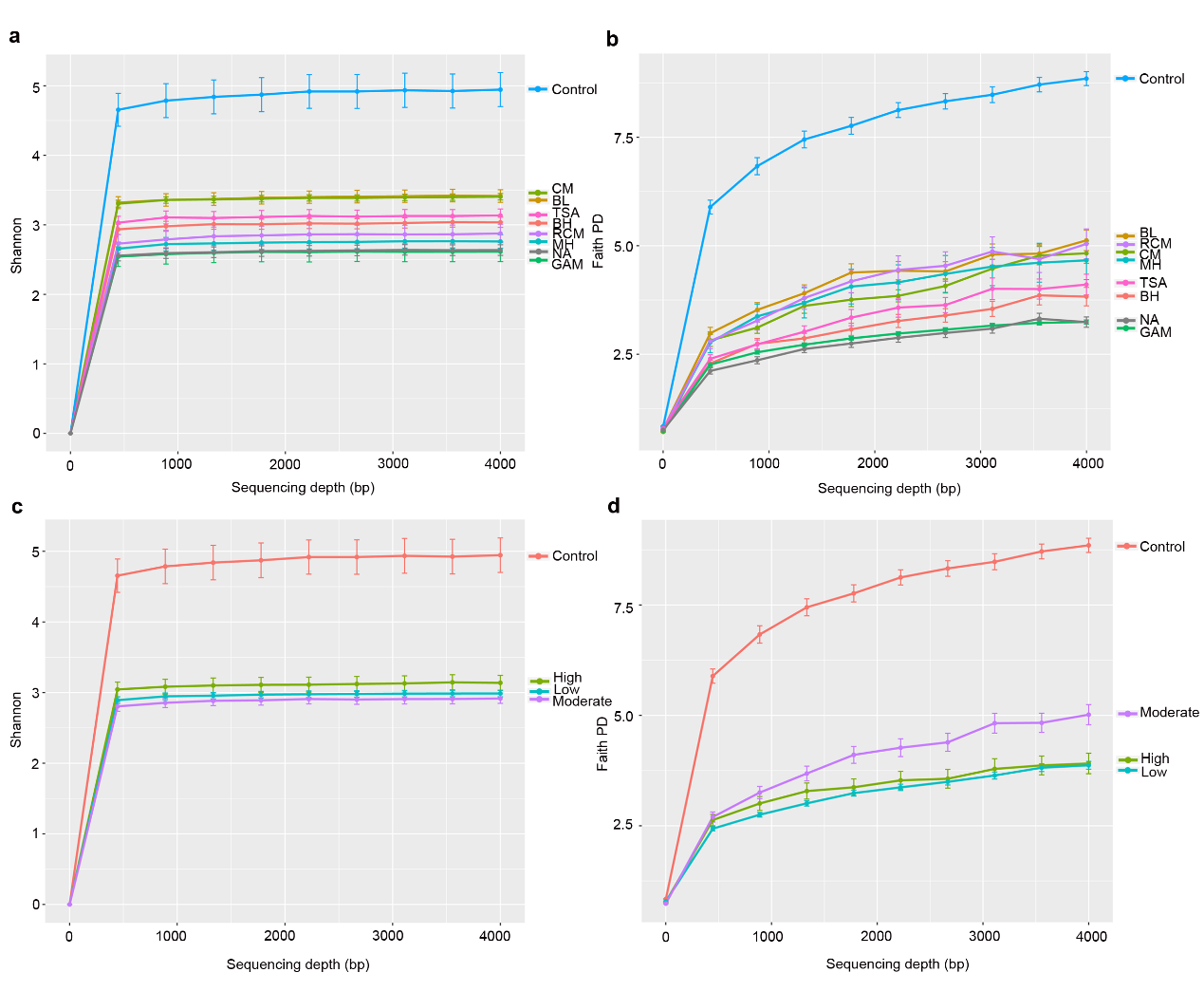


**FIGURE S1.** **Rarefaction curves in all samples.** Rarefaction curves based on Shannon (**a**) and Faith PD (**b**) index in various media. Rarefaction curves based on Shannon (**c**) and Faith PD (**d**) index in low-, moderate-, and high-density group.


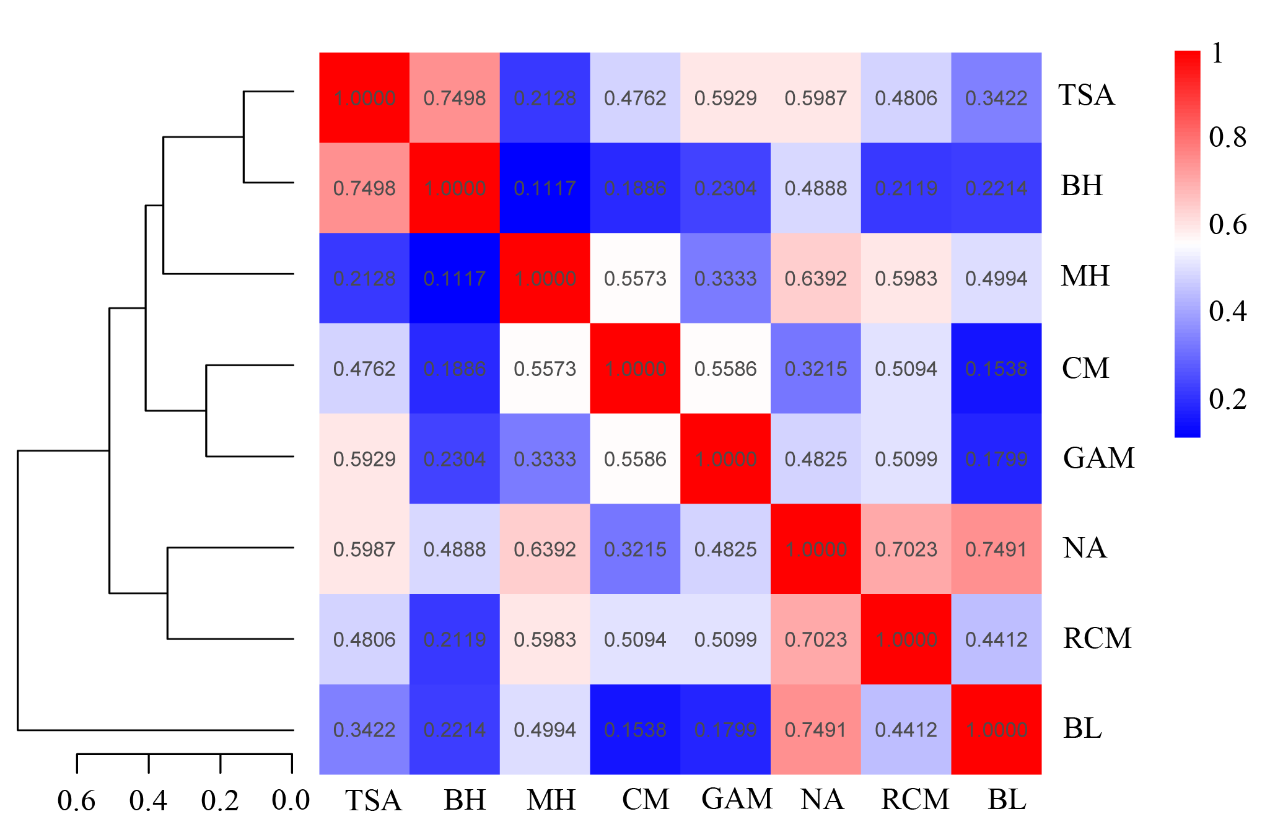


**FIGURE S2. Correlation and cluster analysis of nutritional components in medium based on Spearman correlation coefficient.**
